# Healthcare and Health Problems from the Perspective of Indigenous Population of the Peruvian Amazon: A Qualitative Study

**DOI:** 10.3390/ijerph17217728

**Published:** 2020-10-22

**Authors:** Bárbara Badanta, Giancarlo Lucchetti, Sergio Barrientos-Trigo, Elena Fernández-García, Lorena Tarriño-Concejero, Juan Vega-Escaño, Rocío de Diego-Cordero

**Affiliations:** 1 Research Group PAIDI-CTS 1050 Complex Care, Chronicity and Health Outcomes, Faculty of Nursing, Physiotherapy and Podiatry, University of Seville, 41009 Seville, Spain; bbadanta@us.es (B.B.); sbarrientos@us.es (S.B.-T.); efernandez23@us.es (E.F.-G.); 2 School of Medicine, Universidade Federal de Juiz de Fora, Juiz de Fora 36036-900, Brazil; g.lucchetti@yahoo.com.br; 3 Research Group CTS 1054 “Interventions and Health Care. Red Cross, Spanish Red Cross Nursing School, University of Seville, 41009 Seville, Spain; 4 Research Group CTS 969 Innovation in HealthCare and Social Determinants of Health, Faculty of Nursing, Physiotherapy and Podiatry, University of Seville, 41009 Seville, Spain; rdediego2@us.es

**Keywords:** health inequalities, community-engaged research, culturally appropriate, indigenous, Peruvian Amazon, traditional medicine

## Abstract

Indigenous communities usually experience higher levels of mortality and poorer access to healthcare services compared to non-indigenous communities. This study aims to understand the most prevalent health problems and their treatment in the Asháninka indigenous communities of the Peruvian Amazon. We conducted an ethnographic study in order to explore the perceived health problems, the use of traditional medicine and the resources offered by the official Peruvian healthcare system. Field notes and semi-structured interviews were used. A total of 16 indigenous and four non-indigenous people were interviewed, and interpretative analysis was used to identify themes. The Asháninka community is an overlooked population, which, due to distance restrictions, misconceptions and ethnical disparities, is far away from an appropriate healthcare system and is subjected to acute medical conditions such as infections and gastrointestinal problems. This group tends to seek traditional medicine, mostly herbal medications and traditional healers. The use of a health professional is seen as a last resort. Although the official Peruvian health system incorporates community participation strategies to improve the healthcare of indigenous people, the shortage of material, human resources and cultural sensitivity makes this difficult. Healthcare strategies should be devised and implemented in order to minimize health inequality in this population.

## 1. Introduction

According to official numbers, it is estimated that 372,000 indigenous persons live in the Peruvian Amazon region, representing 51 out of the 55 indigenous groups living in Peru [1]. These indigenous communities suffer more social inequalities than any other group [2], having higher rates of infant mortality rate, lower access to health services, and worse nutritional status compared to the general population [3,4,5]. This remarkable inequality leads to several health problems in this population, such as higher levels of gastrointestinal problems [6], infectious diseases (e.g., tuberculosis) [7] and poor oral health [8].

Cultural factors seem to have a strong influence on these health outcomes and studies have shown that traditional healers are still the primary source of healthcare for most of the indigenous population in the Amazon [9]. These healers tend to use plant-, animal- and mineral-based therapies, spiritual healing and manual techniques in order to treat, diagnose and prevent illnesses in order to maintain the wellbeing of their patients [10]. In a previous study, 83% of the indigenous persons in Loreto (Peru) had used traditional medicine in the previous year and almost 40% had never seen a doctor [11].

Although the use of traditional medicine is common among the indigenous population, the distance to healthcare facilities, a lack of money and a lack of health access provided by the government are the most noted factors that prevent these groups from seeing a doctor [11]. The Peruvian government offers health services to the uninsured population through Comprehensive Health Insurance (Seguro Integral de Salud, SIS) [12]. However, the Peruvian Health System (PHS) has serious difficulties in providing health coverage to the indigenous population. A recent study found that only 64.8% of the rainforest population received medical care compared to 73.1% in the general population [13].

These challenges seem to increase when the indigenous population lives in rural communities with difficult access [14]. Each healthcare facility in Peru has a limited coverage (500 to 800 families) and usually is composed of a physician, an obstetrician, a nurse, nursing technicians and Community Health Agents (CHA). CHAs are usually community persons working as volunteers and are very important figures to make a connection with the traditional healers and to promote treatment for vulnerable populations [15].

All these factors make this population difficult to treat. Therefore, it is important to understand how the indigenous population who live in the Central Jungle of the Peruvian Amazon use their traditional therapies as well as the official health resources to overcome their health problems. It is important to highlight that the Peruvian government does not separate data by ethnicity and, for this reason, little is known about the population living in the rainforest [13]. This is a potential problem, since the rates of health problems and infectious diseases are not reliable [16], making this population overlooked in Latin America.

Therefore, the objective of the current study was to explore and understand the most prevalent health problems and their treatment in the indigenous Asháninka of the Peruvian Amazon. Our research questions were as follows: “What are the main perceived health problems and how does the indigenous Asháninka population address them?”, “What are the main factors associated with the use of Peruvian official healthcare services?”, and “Which barriers for healthcare are reported by the indigenous Peruvian population?”.

## 2. Materials and Methods

### 2.1. Design

A qualitative ethnographic study was conducted in the Amazon region of Peru in South America. We chose a focused ethnographic approach based on Roper and Shapira’s framework (2000) [17], as this allows us to explore a particular topic of interest in a specific context, and to perform the analysis focusing on subcultural groups rather than on entire societies. This approach is characterized by (a) a conceptual orientation provided by a research team; (b) a focus on a discrete community; (c) a focus on a problem within a specific context; (d) a limited number of participants; (e) the use of participants who may hold specific knowledge; and (f) the use of selected episodes of participant observation [18]. Data collection consisted of participant observation, field notes, and semi-structured interviews, all conducted by the principal investigator (BB) over three months in 2019.

### 2.2. Population and Setting

**Figure 1:**
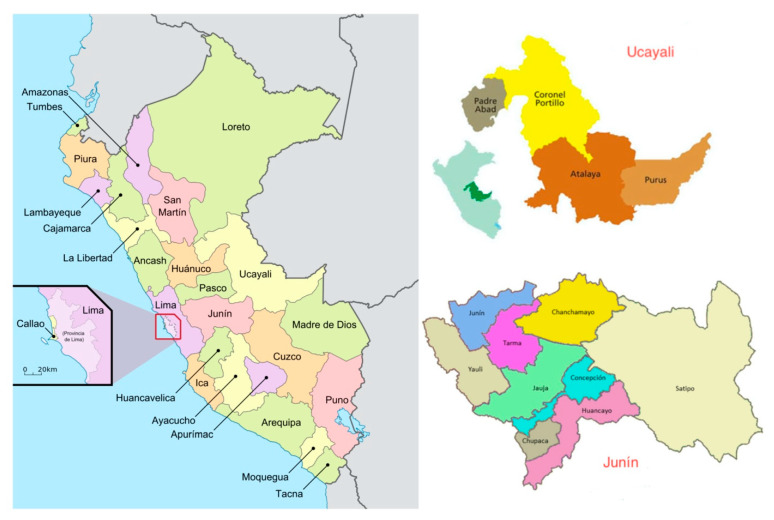
Map of Peru: Central Jungle of the Peruvian Amazon. Junín and Ucayali departments [19,20,21].

Our study took place in the Central Jungle of the Peruvian Amazon, specifically in 10 areas belonging to the Junín and Atalaya provinces (Junín and Ucayali departments), all of them situated in the country of Peru (Figure 1). According to the last Peruvian Census, there are 372,000 indigenous persons living in the Amazon region and the districts of Junín (35,920 people) and Ucayali (35,920 people) represent 16.9% and 17.3% of the indigenous population in Peru, respectively [1]. Inside these districts, the provinces of Llaiya and Nopoki are rural villages with indigenous and settler inhabitants, while the remaining areas (i.e., Shonori, San Pedro de Lagarto, Huantashiri, Shintoriato, San Juan de Cheny, Quimiriki, Bajo Capiri and Maranquiari) are native indigenous communities (CC.NN). For the present study, an indigenous person was defined as “the one that descends from populations that lived in the country before the colonial era. They are organised mainly in native and peasant communities and they generally maintain all or some of the customs and traditions” (National Institute of Statistics and Informatics of Peru, INEI).

Healthcare is mainly offered in rural and community settings. The most basic healthcare assistance is located in the closest areas or districts (i.e., Mazamari and Río Negro), while a more specialised care is offered in Satipo (central health network). The rural medical centers that serve the Asháninka population offer general medicine, pediatrics, obstetrics and dentistry services. In addition, the Llaiya center offers a clinical analysis laboratory.

Five towns or communities can be accessed by vehicle, and only Huantashiri has a rural primary healthcare center within the community. Other communities have their centers relatively close, and the indigenous population must walk between 10 to 45 min to get into these health facilities (Shonori, Shintoriato, San Juan de Cheny, Quimiriki, Bajo Capiri). Finally, people who live in Maranquiari and San Pedro de Lagarto should take a boat in order to get to the nearest healthcare service, taking between 45 and 90 min.

Although the researcher (BB) made an effort to observe most people living in the geographic areas of the study (i.e., children, adults, and older people), the direct participants (i.e., interviewed people) were selected if they were adults (18 years old or more) from the indigenous Asháninka ethnicity, relevant persons in this community and living in the Amazon Peruvian region. The choice of the Asháninka ethnicity lies in the fact that this is the most representative ethnicity in the region (representing a total of 520 communities). Thus, this seems to be an appropriate group to investigate the influence of cultural beliefs on healthcare [22]. Assemblies were held in each community in which, after explaining the objective of this study, its members named the most relevant people in their community, offering supporting arguments. The main researcher respected the majority of votes towards those relevant people and their voluntary acceptance to participate. In the present study, a total of 16 indigenous Asháninka agreed to participate.

In addition to these participants, researchers opted to include other non-indigenous key informants (which provided different perspectives), in order to corroborate the information obtained from indigenous Asháninka. Therefore, the inclusion of other four participants was accepted and in-depth interviews were carried out. The following inclusion criteria were established: living or working in the community, and being considered a relevant adult as determined by indigenous members.

In order to include different perceptions and insights, we included a sample of participants with different profiles concerning sex, place of residence, social characteristics, jobs and ethnicity (Table 1).

### 2.3. Procedure

Purposive and snowball sampling were used to select the participants [23]. They were identified and invited by the main researcher, who is a nurse with expertise in transcultural nursing and health inequalities. She started working in the field of International Development Cooperation in these areas, specifically monitoring and evaluating projects for housing adaptation, agricultural production and child health, identifying health problems for women and girls (all this projects were demanded by the communities themselves). She also met and lived inside some Asháninka communities. In this study, the main researcher maintained a guest role, expressing interest in the traditional approach to health problems, but not serving the community members as a nurse.

A certain educational level or age was not required on behalf of the nurse. The focus was on participants’ shared behaviors and experiences and for this reason, we worked under the assumption that they would share cultural perspectives, even if they did not know each other [17]. In line with a focused ethnographic approach [17,24], observations were performed as part of the main researcher’s everyday work and occurred at selected times of the day, inside the researcher’s communities. Observational data were recorded as handwritten field notes, focusing on common characteristics from all geographic areas, the available health resources, and how the participants dealt with their health problems. All of those aspects were agreed upon and accepted by members of the communities here studied. Field notes included information about witnessed events, verbatim verbal exchanges, and the researcher’s personal interpretations of events. Informal conversations and the interviews allowed the researcher to examine if interpretations of meanings behind observed behavior coincided with participants’ own understanding. Participants were asked to evaluate the material and their responses were analyzed and added.

A total of 20 interviews were carried out face-to-face (one per participant without repetitions), in Spanish, and they lasted 30–60 min. Although Asháninka is the local language of the indigenous participants, Spanish is the official language of Peru, and both the indigenous and non-indigenous participants and the main researcher shared this characteristic. They were audiotaped by the main researcher, transcribed verbatim by two researchers, then translated into English by a translation company. Data collection continued until data saturation was reached.

Informed consent was obtained from all participants and the researcher also obtained the approval of the indigenous community leader, the local non-governmental organization and the public administration of the departments where the study was carried out.

### 2.4. Instrument

The interview script was based on the idea that the most common health problems of this population are related to living conditions and that the indigenous population alternates the use of traditional medicine with official government healthcare. An expert panel, using the Delphi method, carried out an analysis of the content of the script to assess its appropriateness and the collection of data was carried out in the period between January and March 2019 [25]. Assessment of the initial script by the panel was done online in two rounds.

In addition, a pilot testing of the interview guide was done to confirm its coverage and relevance, to test its implementation and to identify the need to reformulate questions. Six indigenous Asháninka (3 men and 3 women) attended for two days and the local Non-Governmental Organization (NGO) that works in the area participated.

The interview script included information on sociodemographic characteristics (indigenous group, sex, place of residence, social and labor profiles and ethnicity). All interviews were conducted using open questions starting with the following: How is the health of people living in your native community? What kinds of diseases are most common among children? And among adults? What factors have an influence on the health problems experienced by the people in your community? What is usually done when a person is sick? How is traditional medicine used? For what kind of problems? How is the official healthcare system accessed? What services are provided to you? Do you experience any barriers to access the official healthcare system?

In the case of non-indigenous respondents, the questions were based on their knowledge concerning the native population they work with.

### 2.5. Data Analysis

The qualitative analysis was carried out following the steps proposed by Braun et al. [26]: (1) familiarity with the data; (2) generation of categories; (3–5) search, review, and definition of themes; and (6) the final report.

Transcription, literal reading, and theoretical manual categorization were performed and the NUDIST Nvivo (version 12, University of Seville, Seville, Spain ) software was used. Data analysis started with a thorough reading of the information collected (field notes and interview transcripts) in order to ascertain an overview of the respondents’ experiences and to gain an understanding of the content. This was done by two researchers. The analysis continued by organizing descriptive labels, focusing on emerging or persistent concepts and similarities/differences in participants’ behaviors and statements. The coded data from each participant were examined and compared with the data from all the other participants to develop categories of meanings. In addition, 2 participants checked the results. They were asked to evaluate material through open-ended responses and their proposals were analyzed and added. Finally, the different categories were gathered (grouped) under two main themes: “Health problems” and “Health practices”. The categorization and coding of the data are shown in Table 2.

### 2.6. Trustworthy

This research followed The Consolidated Criteria for Reporting Qualitative Studies (COREQ) [27]. The methods used for guaranteeing quality were data triangulation, including participants with different sociodemographic characteristics, and the triangulation of data analysis via different researchers (see the Supplementary Material Table S1).

### 2.7. Ethical Considerations

The study was approved by the Andalusian Research Ethics Committee (Code: 1469-N-19). All participants received written and oral information about the study, including the right to withdraw and a guarantee of anonymity. Data were anonymized by removing names/locations and by changing details. Interview transcripts and audiotapes have been kept in locked files. Persons not involved directly in the study were not interviewed or identified in any analysis.

## 3. Results

The native communities of this study are composed of indigenous Asháninka families living in the Central Jungle of the Peruvian Amazon. A common feature of these families is their dedication to agriculture, making their farmland (chacras) their main source of income. Unfortunately, little income is provided by this activity, having a negative impact in the acquisition of uncultivated food. The lack of resources seems to be responsible for the high rates of acute diseases in this population, mainly related to gastrointestinal and pulmonary problems. In order to treat these problems, our participants report that they use traditional therapies, administered by family members or specialized people such as local healers.

### 3.1. Health Problems

Health problems were an important category presented in our analysis. Statements included some factors associated with the prevalence of disease in children and adults (Table 3). Although specific health problems were mentioned, there is a general idea that indigenous persons have a poor health condition (“we are all sick, we are not healthy”) and that their economic situation may interfere with healthcare access and the proper treatment of these diseases.

Participants (both indigenous and non-indigenous participants) have agreed that gastrointestinal infections and respiratory problems (i.e., flu, sore throat and runny nose, that occur mainly in the winter) are important problems in the indigenous community. In the case of children, they often suffer from colds, pneumonia and fever complaints, the latter being recognized by indigenous mothers as a disease. All these respiratory infections seem to be related to the low vaccination rates and the poor housing conditions (i.e., houses are not adequate for the climate conditions faced in the rainforest).

Gastrointestinal infections also appear frequently in most statements and are mostly linked to the poor sanitation and the lack of hygiene. The first author observed that, when indigenous persons work in the cocoa plantations, they commonly eat fruits by bringing their hands to their mouth without washing them. Another important problem reported by health professionals was the fact that indigenous communities did not have treated water. In fact, there were no water chlorination systems nor storage reservoirs in this region and the indigenous persons chlorinated their water only when the municipality provided them with the resources. Likewise, few people boiled water for consumption and there was a lack of information concerning the contamination of water. As an example, the principal investigator commonly observed that indigenous persons used water from the same spot where they showered themselves or washed clothes.

Due to this lack of hygiene, high rates of malnutrition and chronic childhood anemia are also observed in children, probably as a consequence of the constant diarrheal episodes and certain nutritional deficits (I-8; non-indigenous, woman, pediatrician) “We have achieved a 50% reduction in malnutrition, but I must recognize that more than a year ago, it affected 100% of the children in this community”. During participation in public health campaigns in three communities, the principal investigator observed that parameters such as height, weight, and blood hemoglobin levels were all lower than expected in the pediatric group.

Other sources of infection noted in these communities were caused by bacteria (e.g., cystitis), insects and parasites. In most situations, a lack of hygiene was considered to be responsible for the infection: (I-7; non-indigenous, woman, doctor) “People lack personal hygiene, they don’t bathe much. I check them and sometimes the pant is not clean, or there is a rotten smell”; (I-10; non-indigenous, woman, doctor) “Sometimes the population itself is not aware that hygienic conditions can be the source of health problems, they think they have a virus but not that it is due to their hygiene”.

All health professionals interviewed agree that skin infections are also common problems in these communities. Scabies are associated with the overcrowding of people in homes, and leishmaniasis is linked to the presence of insects and the absence of preventive measures, such as repellents or mosquito nets. In general, houses were built with low-durability materials (i.e., wooden slats with openings between them that allow the entry of vectors and water) and these conditions perpetuate the presence of mosquitos.

Other prominent health problems are musculoskeletal, unwanted pregnancies and cancer. Musculoskeletal conditions among adults are attributed to daily work activities carried out for years, with forced postures that increase the risk of bone pain and contractures. Unintended pregnancies are another remarkable problem, being reported even in 11-year-old girls, and complications during childbirth and vaginal tears are also commonly observed. The poor prenatal control and the hygienic conditions in which childbirth takes place could be related to some cases of neonatal sepsis attended by the health staff.

Cancer, sometimes called “lumps” by indigenous people, also begins to affect these communities. Specifically, health professionals mention cases of prostate, stomach or breast cancer. In the Shonori community, the healer talked about her newly diagnosed breast cancer: (I-3; indigenous, woman, healer) “It hurts this part of my breast and a doctor told me to take a radiograph and that I have a principle of small malignant tumor. I noticed it because especially when working, it hurts a lot, when I take the machete”.

The indigenous population also mentions “non-physical” health problems, known as “spiritual problems”. Different girls and the coordinator of a residence for students reported continuous episodes of spiritual suffering that affected several of their partners: (I-9; indigenous, woman, coordinator or a residence for students) “She was as if awake, but sleeping. She said she saw a person who was suffering and wanted to kill her. Some men came and could not control her. This started with a girl, and then with another and another. They say that sometimes this person still chases them and they see him and they get scared”.

### 3.2. Health Practices

The information obtained from the participants’ observation, informal conversations and interviews have allowed the researchers to understand the different “levels of care” that are used by the indigenous communities to address their health problems. The first level of care in this community is the use of traditional medicine, which can be homemade or provided by a specific healer. In the case of an unsuccessful treatment, participants search for the second level of care, which is provided by the health professionals of the official Peruvian healthcare system.

#### 3.2.1. Traditional Medicine

As reported previously, the first level of care for the indigenous community is the use of traditional medicine. Indigenous persons tend to use plants as the first level treatment, but the types and amount of plants used can vary significantly according to the community studied. While some medicinal plants are used as crushed ointments or poultices (e.g., chupasangre against bumps or the leaf of tobacco for wound healing), other seeds are ingested, such as yahuar piri piri for the treatment of intestinal infections and bloody diarrhea. Another method of preparation is boiling in water, as in the case of oregano for heavy digestion or stomach pain. Some of them may even have different application options, such as sangre de grado: (I-12; indigenous, woman, secretary) “tears serve for all inflammations and healing. It is also taken for the gut, as antiseptics, for operations. You can apply directly or drink 2 or 3 drops in a glass of water, no more because it is very strong”.

In other cases, traditional medicine could also include animal remedies. This is the case of the gall of the majaz (large rat of the bush), whose use for a year provides contraceptive protection. Other animals are used as antidotes; for example, the river shrimp (camarón de rio) for scorpion stings or the termite comején for certain butterflies’ bites.

In addition to the physical effects of traditional medicine, participants have reported that there is a “spiritual component” in these plants that could enhance the treatment: (I-13; non-indigenous, man, priest) “there are plants that have a meaning that may seem strange, it’s what I call spiritual magic, but it really works. I have checked it and I have seen it with a man who was bitten by a poisonous viper”.

(I-2; indigenous, woman, community leader) “When we are worried or they say you have “mal aire (evil eye) [“Mal aire (evil eye)” is a metaphysical disease, whose terminology has been used since ancient times. The Indigenous population describe it as a phenomenon that occurs when a person sees someone walking at night and a light wind blows and this is accompanied by some spectrum. That ghostly appearance is considered an intrusion of evil since it enters the body of the subject who walks there and makes him sick. Sometimes it manifests itself in strange acute diseases, such as reddish and watery eyes and vomiting after encountering a spectral being or walking through places where there is bad energy], when you don’t have a job or you don’t feel good at job, you have mala vibra [bad vibes, bad energy]; when we are alone, we hear things, or imagine that someone is calling us, we take leaves, we boil them and we bathe. That’s how you feel well”.

(I-12; indigenous, woman, secretary) “My husband died and I saw him walking, but not in person like us, but his shadow. I started to vomit and if I didn’t take the piri piri I would have died. To stop seeing this shadow, I also put the crushed internal seeds in my eye (placed in a rag to make it wet)”.

In fact, although these spiritual issues are prevalent in this society, this is a universal truth found in every culture across time and geography and in no sense a unique observation among this ethnic group. Given these mystical issues, the indigenous population believes that the official health system is limited and does not offer them the services they consider appropriate for these spiritual problems, and, therefore, traditional medicine is considered as the only effective resource. As an example, the governmental health system has no treatment for “mal aire”, so indigenous people use their own treatments: (I-3; indigenous, woman, healer) “We use our resources [traditional medicine] in the case of mal aire (evil eye), scare or witchcraft, because they can do nothing or do not believe it [the official health system]”.

##### Self-Care at Home

The observations provide information on the important role of women in the healthcare of their children. Women are responsible for planting the medicinal plants, collecting information concerning the use of traditional medicine and treating the other members of the family: (I-15; indigenous, man, cocoa producer) “My mother [older woman] has planted there; she knows what are the healing herbs and to stop bleeding”. It is observed that women in the communities of Shonori and Bajo Capiri plant and take care of small orchards of medicinal plants next to their home. Each woman plants those herbs that can be used as a remedy more frequently, such is the case of piri piri for cuts. In the event that a woman does not have a certain plant, she usually receives it from another neighbor in her community.

Not only are women aware of the effects of natural remedies, but they also provide accurate information on use, dosage, duration of treatment or adverse events (Table 4).

##### Traditional Specialized Care

When the homemade remedies do not work and the health problem persists or worsens, participants search for another type of treatment, considered a more specialized type of care which is provided by a traditional healer. Even when this specialized agent is not available in one community, it is sought in another community nearby. This was clearly observed by the principal researcher while investigating a residence with young girls. These girls refused to go to the hospital and demanded the presence of the head of an indigenous association along with a spiritualist healer.

The healers, also named shamans, are specialized indigenous persons who received at least one year of training. They are responsible for addressing the health problems of the indigenous population and use medicinal plants and spiritual rituals to treat patients. One of the best known rituals is the use of Ayahuasca, a tree from which the healer makes a drink to obtain spiritual and bodily healing: (I-14; indigenous, man, project technician) “If you want to know if someone wishes you evil, you can take the Ayahuasca. You start to see things, scenes, and with the indications of the healer you can know who is wrong”.

(I-1; indigenous, woman, university professor) “I was in poor health. I bathed, took pills and put on cream but nothing. On recommendation I went to a healer and did the Ayahuasca ritual. I saw how a house was built and crumbled, I built my boat and it also split. This was what happened in my life, that everything fell and nothing went well. Ants and a giant worm came out of my body. These bugs that represented evil began to leave my body with the healer’s song; I was being cured and since then everything improved”.

#### 3.2.2. Official Health System

##### Use of the Official Health System

The fact that the indigenous population is recognized in Peru as a group in poverty, living in a vulnerable situation, the government confers the use of SIS (basic medical insurance) to this group. However, the official health system, as mentioned previously, is a resource that the indigenous population tend to use only when the traditional medicine is not enough to solve the problem: (I-5; indigenous, woman, traditional midwife) “When I see that it cannot be done at home or there will be problems [referring to childbirth], I say to the woman: I can’t see you, go to the doctor”. Nevertheless, even while using the official health system, traditional therapies are still used by the indigenous persons as a combined therapy. Thus, although medications such as monthly injectable contraceptives, antipyretics, anti-inflammatories and analgesics are used to treat acute medical conditions, these drugs are discontinued when the symptoms disappear: (I-7; non-indigenous, woman, doctor) “They usually come after 2 or 3 months, because they want to follow their customs and heal with their herbs. That is why they do not usually follow medical controls”.

On the other hand, they do not trust the use of official health services due to uncertainty about the true fate of blood taken in analytics, the beliefs that vaccines will make them sick (I-11; indigenous, woman, community health agent), and the perception of rejection by health professionals: (I-9; indigenous, woman, coordinator of a residence for students) “The herbs that women take in childbirth are strong and for them (health personnel) they stink”; (I-20; indigenous, woman, JASS member) “Although they [doctors] know that we take plants, after the surgery, they don’t say to use them to heal you”.

##### Strategies to Enhance Healthcare

To allow better access to certain health services and to reduce the economic costs to indigenous persons, the health facilities use medical specialists to perform more specialized care when needed: (I-10; non-indigenous, woman, doctor) “Sometimes we get some doctors from Lima to come; in October, ophthalmologists will come to operate cataracts using lasers in adults and the elderly”.

(I-7; non-indigenous, woman, doctor) “When we detect that several patients require psychological service, we ask for support from “Mazamari”. We will have the next psychological attention within 10 days”.

Another important strategy to minimize these cultural differences between the official health system and the indigenous communities is the use of CHAs. CHAs are important and influential people within their own community that work in a multidisciplinary team performing advisory functions, family assessments, child health monitoring, and health education. The principal investigator was able to observe the work of CHA in the community, including educational workshops on correct hand washing or proper incorporation of local foods to achieve an optimal nutritional balance in children. The indigenous Asháninka people spoke with greater confidence with these agents, they allowed them to enter their houses and this allowed them to alert the doctor to vital emergency situations, one of which even saved the life of a baby.

(I-11; indigenous, woman, community health agent) “We are seven CHA working with 60 moms. We usually walk from an hour to an hour and a half doing home visits. We record how mothers are taking care of their children and assess the conditions of their homes. We can enter their lives because we are people closest to them”.

Another strategy to improve healthcare is a system of financial compensation for mothers, which meets the minimum requirements for their children. The government program is named “Chispitas” and aims to monitor the nutritional status of children (I-10; non-indigenous, woman, doctor). There is also the social support program named “Juntos” [Together], in which every child receives routine health check-ups, receives the correct doses of vaccination and has their school attendance verified (I-6; indigenous, man, agricultural technician).

##### Weaknesses/Barriers to Healthcare

The most common barriers pointed out by participants were the difficult access to healthcare and the low coverage of the health system. Complex medical conditions are treated only in central areas such as Huancayo (7 h away from Satipo) or the capital of the country, Lima (12 h away from Satipo). This distance implies in the temporary abandonment of work activity and unexpected economic costs that Asháninka families usually cannot afford: (I-4; indigenous, woman, JASS member) “I have to go to Huancayo and would have to pay the ticket, the paperwork for the ticket, and also the stay and the operation. We will die because we have no money”.

Although medical facilities near the indigenous communities offer basic healthcare services, several deficiencies were noted by the principal investigator. It was observed that the facilities were old and the material was damaged and incomplete. During the visit, one of the medical facilities was running out of water for a week: (I-17; indigenous, woman, community health agent) “We have the support of the municipality, but there is no stretcher, nor equipped briefcases”. It was also common to hear health workers saying that some products (e.g., hand soap) were bought by themselves or by non-governmental organizations.

The lack of health personnel was another problem in the provision of care to these patients. As a result, professionals had to perform jobs even without having the proper competence to do so, such as cleaning, administrative tasks and treating health conditions outside their area of expertise: (I-7; non-indigenous, woman doctor) “If I’m not there, the nurse technician will attend the patient. I know it’s wrong, but there is no other doctor. The day I rest there is no doctor, and the same happens with the dentist for example”. Another problem is that the working conditions do not favor the continuity of care. Although in-training physicians are obliged to complete rural service during their studies, all of them leave the job after one year and new doctors have to be selected.

The fact that there is a conflict between medical personnel and indigenous persons due to cultural issues creates further difficulties. The health professionals interviewed recognized that the traditional medicine of the Asháninka population deserves respect, but they have no knowledge about these remedies: (I-7; non-indigenous, woman doctor) “I can’t lie to you, I don’t have much notion of herbs and their properties. They have so much diversity that I know them when they come and they tell me”. However, when the healthcare staff know their properties, they try to include these elements in the treatment options. An example the doctor from Huantashiri, who included sacha inchi as a treatment against a patient’s hypertriglyceridemia: (I-10; non-indigenous, woman, doctor) “it helps them to increase their level of good fats such as HDL. As a start, I do not suggest western drugs, but their products, which are healthy and available”.

It is this lack of knowledge about popular ways of addressing health problems that makes the connection between the traditional and official health system difficult: (I-16; indigenous, man, community leader) “There is a native local midwife, but the health staff does not have much contact with her. Sometimes it is not known that a woman is pregnant or has given birth. Nobody alerts health professionals”.

## 4. Discussion

The findings of the present study indicated that this Peruvian Amazon indigenous community is subjected to several acute health problems and uses mostly traditional medicine to treat them, relying on health professionals only as a last resort. The most common health problems identified in this study were gastrointestinal and respiratory infections.

Concerning respiratory problems, there is an increase in respiratory conditions during the “slash-and-burn” season [28], due to the agricultural activity that characterizes these communities. Likewise, there is also a low rate of vaccination in this population [4,29], mostly justified by distrust on pharmaceutical medications [30]. In relation to the gastrointestinal problems, the lack of hand washing and consumption of untreated water are the most influential factors for acute diarrhea according to Morocho and Espinoza [31]. Handwashing was almost non-existent during work on the chacras and this is due to the following reasons: limited access to water, lack of knowledge of the benefits of handwashing and lack of access to soap (i.e., they use only water to wash their hands) [32].

Another important health problem in our sample was child malnutrition. In previous studies, the prevalence of chronic malnutrition (56.2% versus 21.9%) and anemia (51.3% versus 40.9%) was higher in the indigenous population compared to the non-indigenous population [4], which supports our qualitative findings. One reason could be the fact that there are strict government maternal–child health policies where mothers whose children do not reach adequate percentiles may be denied access to health services in the future, which includes the provision of monthly food rations [33].

Indigenous persons clearly search for the traditional medicine as a first attempt to heal their health problems. The first level of care is provided by women’s homemade remedies or by traditional healers and, if the symptoms persist, the second level of care (official Health system) is then used. Although the use of this type of traditional medicine is a characteristic of this ethnicity, it is important to note that seeing a clinician as a last resort is quite common across cultures, in a sense that individuals tend to treat themselves, ask friends and even search the internet before considering specialized care.

While analyzing the statements of the participants, it is clear that indigenous people tend to combine both treatments. This dual approach seems to be a good option for public health managers, since people know their ways are respected and do not have to choose one system over the other, reducing the suspicion of Western medicine. However, in some circumstances, if a qualified physician fails to cure the patient, they return solely to traditional healers [34]. These healers use a high number of herbal remedies to treat several conditions, such as musculoskeletal disorders, gastrointestinal complaints and skin conditions [35] and the use of traditional healers is widely disseminated in Peruvian society and specifically among indigenous people, who even use their knowledge as a source of income with non-indigenous customers [36]. The fact that Western medicine cannot guarantee curative treatment in pathologies like cancer could explain the results of a study conducted among Peruvian patients with hepatic tumors. In this study, 68.3% of the people interviewed claimed to use Herbal Medicine on a regular basis for general health preservation based on the advice and recommendation of their nuclear family, friends, and neighbors [37].

Current studies with indigenous populations confirm the results of this study on the use of official health services. Limited access to health information, distrust of health providers and services, and limited cultural responsivity among non-Indigenous clinicians, are determining factors in health and care [38]. Good strategies to minimize the cultural differences between indigenous persons and health personnel are the use of local materials in the construction of health facilities, the use of indigenous language to alert people to these spaces and the provision of specific spaces for the practice of traditional medicine [39]. The CHA also has an important role in this relationship and it should be acknowledged. CHAs have an important relationship with the indigenous population and could promote strong ties between health professionals and the community, favoring the adherence to treatment and improving preventive strategies of care.

Despite all the cultural factors, the workforce is also limited in these regions and Peru lacks systematic policies for attracting and retaining these human resources. Incentives could be used to end this problem in remote areas such as higher wages, opportunities for further training, longer/permanent contracts, better infrastructure and medical equipment, more staff, and recognition by the health system [40]. As an example of this inequality, the most common reason for the limited health screening campaign for the indigenous population was the lack of budget (59.3%) followed by the lack of human resources [41].

As previously reported, the indigenous group is a very vulnerable population, and their health needs exceed the capacity of existing healthcare services in Peru. To reduce health disparities due to their cultural beliefs and their approach to health and wellness, it is paramount to address intercultural and participative aspects of healthcare models [42]. These are the biggest challenges for the health administrators of the Peruvian government. In a recent review [43], the authors reported some of the most important healthcare strategies used for indigenous groups. Some examples include improving essential infrastructure (healthcare facilities in remote areas), increasing access to education, enhancing cultural sensitivity for healthcare professionals, incentives for healthcare professionals, the collaboration of indigenous communities with universities, and improving social accessibility, among others.

### Study Limitations and Strengths

This study has some limitations and strengths. It is an innovative piece of research, since it has included the point of view of the indigenous population from 10 different native areas and communities in the Central Jungle of the Peruvian Amazon, exploring the perceptions of the participants through the qualitative methodology. However, purposive and snowball sampling were used and the generalizability of the conclusions is limited due to the relatively small sample. Therefore, the study should be extended, using a representative sample of different communities in Peru and elsewhere. Likewise, participants were selected in order to represent all community members of these indigenous societies. Therefore, the population was composed of cocoa producers, community leaders, healers, professors, healthcare professionals and a midwife, with the aim of providing an overview of the opinions and perceptions of the community. However, it is possible that some of their views may be different from the views of other community members.

Based on this study, we suggest a few directions for future research. First, the understanding of the gap between health professionals and the indigenous community should be better explored. Second, the investigation of the spiritual needs of this population and their relationship with their treatment should be expanded. Third, the role of CHAs in the health system deserves more attention and should be investigated in future studies, since these professionals are key components of the health system in this vulnerable environment. Finally, it is important to further understand the opinions of health administrators and contrast these opinions against the perceptions of indigenous groups.

## 5. Conclusions

This qualitative study has added to the current literature describing the reality faced by the indigenous population of the Peruvian Amazon. This is an overlooked population that, due to distance restrictions, misconceptions and ethnicity barriers, is far away from an appropriate healthcare system and is subjected to acute medical conditions such as infections and gastrointestinal problems. In order to cope with their diseases, this group tends to seek traditional medicine, mostly herbal medications and traditional healers. Women are mostly responsible for treating the family and the use of health professionals is considered a “next resource” as a process of escalation as their condition progresses.

Although the official Peruvian Health System incorporates community participation strategies to improve the healthcare of indigenous people, the shortage of material, human resources and cultural sensitivity make this attention difficult. Although the role of CHAs is beyond the scope of this study, in our opinion, they can be considered an important group in this healthcare system, decreasing the distance between health professionals and indigenous persons, improving the adherence to treatment and minimizing the health inequality in this population.

## Figures and Tables

**Table 1:** Demographic details of participants.

Code	Indigenous ***	Sex	Place of Residence	Social/Labor Profile
I-1	Yes	Woman	Nopoki (Atalaya)	University professor
I-2	Yes	Woman	Shonori	Community leader
I-3	Yes	Woman	Shonori	Healer
I-4	Yes	Woman	Shonori	JASS member *
I-5	Yes	Woman	Quimiriki	Traditional midwife
I-6	Yes	Man	Llaiya	Agricultural Technician
I-7	No	Woman	Llaiya	Doctor
I-8	No	Woman	Llaiya	Pediatrician
I-9	Yes	Woman	Satipo (Junín)	Coordinator of a residence for students
I-10	No	Woman	Huantashiri	Doctor
I-11	Yes	Woman	Huantashiri	CHA **
I-12	Yes	Woman	San Pedro de Lagarto	Secretary
I-13	No	Man	San Pedro de Lagarto	Priest
I-14	Yes	Man	San Pedro de Lagarto	Project technician
I-15	Yes	Man	San Juan de Cheny	Cocoa producer
I-16	Yes	Man	Maranquiari	Community leader
I-17	Yes	Woman	Maranquiari	CHA **
I-18	Yes	Woman	Bajo Capiri	Agricultural Technician
I-19	Yes	Man	Bajo Capiri	Community leader
I-20	Yes	Woman	Shintoriato	JASS member *

* JASS: Sanitation Services Administration Board (Junta de Administración de Servicios de Saneamiento), ** CHA: Community Health Agents, *** Indigenous: An indigenous people is one that descends from populations that lived in the country before the colonial era. They are organised mainly in native and peasant communities and they generally maintain all or some of the customs and traditions (National Institute of Statistics and Informatics of Peru, INEI).

**Table 2:** Emergent themes, categories and subcategories.

Theme	Category	Subcategory	Description
Health Problems	Health Problems	Diseases diagnosed Signs/symptoms	The health problems reflect alterations of a bio–psychosocial–spiritual nature identified by the indigenous population and health professionals. In addition to signs and symptoms, perceptions of the causes of health problems or certain factors that contribute to perpetuating poor health conditions are also incorporated.
	Attributed Related Factors	-	
Health Practices	Traditional care	Self-care at home Traditional specialized care	The use of traditional medicine includes all those popular health practices, preventive, curative or palliative derived from beliefs, customs and ancestral knowledge, not associated with the usual practices of care of Westernized health systems. It also includes care providers, either at home or outside, such as specialized health agents (e.g., healer, midwife or huesero *). Health professionals have a perception about the health problems of the indigenous population and their methods of approaching these issues. The official health system uses strategic mechanisms to care for indigenous people and faces weaknesses that make care difficult.
	Official health system	Use of the official health system Strategies for attention Weaknesses/barriers to healthcare	

* This is a traditional healthcare provider specialized in the treatment of musculoskeletal system problems. The professionals of the Peruvian health system attribute similar characteristics to that of the traumatologist in Western medicine.

**Table 3:** Main health problems perceived and related factors by indigenous people and health professionals.

Health Problems	Statements (n)	Attributed Causes/Related Factors
**Respiratory problems**	16	I-2; indigenous, woman, community leader “We get the flu from people who come from other places, but we don’t take care of ourselves either”. I-4; indigenous, woman, JASS member “When it rains we get wet and because of the distance we have to walk to the post, our body heats up and we get the flu. I was until last week almost 15 days *wrong*”. I-7; non-indigenous, woman, doctor “We also cannot improve the situation of respiratory infections if we do not have the influenza vaccine for adults”. I-17; indigenous, woman, community health agent “We all needed housing here, when the wind blows strong our entire roof flies and the rain wets our bed”. I-8; non-indigenous, woman, pediatrician “There is quite a lot of overcrowding, they all live together with their little animals inside, they cook with coal and their room is next door. There is no smoke elimination, so that in the long term will affect them with respiratory infections”.
**Gastrointestinal infections**	15	I-18; indigenous, woman, agricultural technician “Sometimes we work in the farm with the machete and when we rest we grab our coconut (refered to using the same machete) and of course, this is how gastritis appears”. I-11; indigenous, woman, community health agent “We are improving on not consuming cold water [referred to water without boiling] because it contains several parasites, so sometimes children get sick from the belly”.
**Malnutrition and childhood anemia**	11	I-20; indigenous, woman, JASS member “Fish farming is not constant; neither is the daily protein consumption or that of other necessary nutrients habitual”.
**Musculoskeletal problems**	8	I-7; non-indigenous, woman, doctor “Due to continuous work in the farms, older patients have muscle contractures and arthralgic problems. I tell them they have to rest but they tell me they can’t because they eat that” (they feed from that).
**Other health problems**	Oral: 4 Ophthalmological: 4 Teenage pregnancies: 7 Injuries, accidents: 5	I-6; indigenous, man, agricultural technician “Everyone here has teeth problems. The dentist says there are some special toothbrushes for babies, but they cost expensive and we can’t afford it”. I-10; non-indigenous, woman, doctor “I detect quite a lot of cataract problems, because the sun is very strong here and it affects a lot, so I am warning them and they are spreading the word. But when they come I find them wrong because nobody tells you anything, they think it’s normal with age”. I-19; indigenous, woman, JASS member “The last time I went to the doctor was because I cut myself with a knife working on the farm. They had to sew my hand”.

**Table 4:** Traditional remedies by indigenous Asháninka women.

Participant (Code)	Verbatim
I-11; community health agent	The achiote is very good for the eyes. Recently we had an epidemic of conjunctivitis. You break the seed and put it in a glass of water for 5 min. A drop appears and you must put it in the eye to relieve. With one or two drops is enough”.
I-4; JASS member	“With a catawa seed you have enough. The inner part is eaten, about half. You should do it on an empty stomach and, you should drink plenty of lemon water, since you can die from dehydration. It starts to sound and turn the belly and you go to the bathroom with diarrhea expelling the parasites”.
I-18; agricultural technician	“Red pinion: one of them is used to heal wounds (the resin) and another is crushed when you have severe headache, the leaves are crushed well and put on as a poultice. And there are also people with beliefs when you have itchy feet, or you get something weird like a tumor and it does not heal with anything ... it also serves as sorcery, but it has to be colored, the white pinion with the green leaves does not work. Be careful because only the sheet is used; The healthy leaf but the seed is poison”.
I-12; secretary	I have faith in this plant, it is the piripiri. Sometimes for body aches, internal fever, for the flu, or “mal aire” [evil eye], squeeze the leaves and heat the water, and bathe with it. You have to meet the doses and finish all that pot bathing, 2 or 3 times a day. My children have grown up with this and it is very effective”.
I-3; healer	If a snake bites you, you should put on the tear that comes out of the catawa’s bark, also called camana [Asháninka term]. Where you have the scar, you should wash it with that, three times a day, and although it takes about 15 days, in the end it heals. In addition to the tear you can use this remedy also as a bath.
I-20; JASS member	I am taking a medicinal herb from the bush to avoid pregnancy. It is a rope that is crushed and boiled and you have to take it after menstruating for three months. And you shouldn’t be close to your husband for it to take effect. If you want another baby, you have to take another herb to end that effect. These herbs are not taken at a young age because it hurts, they are taken from 18–20 years onwards.
I-5; traditional midwife	To avoid problems during pregnancy, you cannot eat beef, because the baby will grow too much. To avoid excessive pain in childbirth, we recommend avoiding the consumption of jungle animals. When the woman begins with labor pains, we know how to control it with herbs, and when the baby is born, we give the woman another herb so that the placenta comes out quickly. Everything has a process and must be given at the right time.
I-1; university professor	When the child is scared because he has fallen or a dog has attacked him, at night he gets up, his emotional stability is not right. For that the cow horn is used; scratches and dust is thrown into a candle. Smoke is the treatment of the child, who gets on top to receive that smoke or steam. The child is passed two to four times through that candle. You do not need a specialist for this treatment, the mother can do it alone. It doesn’t have much risk, you just have to control the candle well.

## Supplementary Materials

The following are available online at https://www.mdpi.com/1660-4601/17/21/7728/s1. Table S1: Consolidated criteria for reporting qualitative studies (COREQ): 32-item checklist
