# Supplementary material for: Healthcare and Health Problems from the Perspective of Indigenous Population of the Peruvian Amazon: A Qualitative Study
Source: Int J Environ Res Public Health. 2020 Oct 22;17(21):7728. doi: 10.3390/ijerph17217728 (PMC7672556; doi:10.3390/ijerph17217728)
Supplement: Supplementary file 1 [file ijerph-17-07728-s001.zip › ijerph-953034-supplementary.docx]

**Table S1**. Consolidated criteria for reporting qualitative studies (COREQ): 32-item checklist

| No | Item | Guide questions/description | Response |
| --- | --- | --- | --- |
| Domain 1: Research team and reﬂexivity | | | |
| Personal Characteristics | | | |
| 1. | Interviewer/facilitator | Which author/s conducted the interview or focus group? | All the interviews were conducted by the main author, BB. |
| 2. | Credentials | What were the researcher’s credentials?  E.g. PhD, MD | BB, SBT, EFG, RDC and GL were PhD. JVE and LTC was PhD student. |
| 3. | Occupation | What was their occupation at the time of the study? | Researcher’s occupations at the time of the study: research professor. |
| 4. | Gender | Was the researcher male or female? | BB, LTC, EFG and RDC were females. JVE, SBT and GL were males. |
| 5. | Experience and training | What experience or training did the researcher have? | All researchers had experience in carrying out qualitative research. BB has been trained to conduct interviews and RD has training in social research. |
| Relationship with participants | | | |
| 6. | Relationship established | Was a relationship established prior to study commencement? | No, there wasn’t. |
| 7. | Participant knowledge of the interviewer | What did the participants know about the researcher? e.g. personal goals, reasons for doing the research | Name, occupation, reasons for doing the research. |
| 8. | Interviewer characteristics | What characteristics were reported about the interviewer/facilitator? e.g. Bias, assumptions, reasons and interests in the research topic | Name, occupation, contact method, reasons for doing the research. |
| Domain 2: Study design | | | |
| Theoretical framework | | | |
| 9. | Methodological orientation and Theory | What methodological orientation was stated to underpin the study? e.g. grounded theory, discourse analysis, ethnography, phenomenology, content analysis | Ethnographic approach with a discourse and content analysis. |
| Participant selection | | | |
| 10. | Sampling | How were participants selected? e.g. purposive, convenience, consecutive, snowball | Purposive and snowball sampling. |
| 11. | Method of approach | How were participants approached? e.g. face-to-face, telephone, mail, email | Face to face. |
| 12. | Sample size | How many participants were in the study? | 16 indigenous Asháninka, and other 4 non-indigenous key informants |
| 13. | Non-participation | How many people refused to participate or dropped out? Reasons? | None |
| Setting | | | |
| 14. | Setting of data collection | Where was the data collected? e.g. home, clinic, workplace | Mainly in workplace and home, and other quiet and comfortable place chosen by the participant. |
| 15. | Presence of non- participants | Was anyone else present besides the participants and researchers? | There were other workers or family members (children among them). |
| 16. | Description of sample | What are the important characteristics of the sample? e.g. demographic data, date | 16 indigenous Asháninka (11 females and 5 males). 4 non-indigenous people (3 health professionals and 1 priest). |
| Data collection | | | |
| 17. | Interview guide | Were questions, prompts, guides provided by the authors? Was it pilot tested? | Yes, they were. / Yes, it was. |
| 18. | Repeat interviews | Were repeat inter views carried out? If yes, how many? | No, they weren’t. |
| 19. | Audio/visual recording | Did the research use audio or visual recording to collect the data? | Audio recording. |
| 20. | Field notes | Were ﬁeld notes made during and/or after the interview or focus group? | Yes, they were (field notes). |
| 21. | Duration | What was the duration of the inter views or focus group? | Average 30-60 minutes |
| 22. | Data saturation | Was data saturation discussed? | Yes, it was. |
| 23. | Transcripts returned | Were transcripts returned to participants for comment and/or correction? | Reviewed by 2 participants. |
| Doman 3: Analysis and ﬁndings | | | |
| Data analysis | | | |
| 24. | Number of data coders | How many data coders coded the data? | Three (BB, RDC and SBT). |
| 25. | Description of the coding tree | Did authors provide a description of the coding tree? | Yes, we did. |
| 26. | Derivation of themes | Were themes identiﬁed in advance or derived from the data? | Themes were derived using both methods. |
| 27. | Software | What software, if applicable, was used to manage the data? | NUDIST Nvivo 12. |
| 28. | Participant checking | Did participants provide feedback on the ﬁndings? | Reviewed by 2 participants (one of them was an indigenous Asháninka, and the another one was a non- indigenous health professional). |
| Reporting | | | |
| 29. | Quotations presented | Were participant quotations presented to illustrate the themes/ﬁndings? Was each quotation identiﬁed? e.g. participant number | Yes, there were. / Yes, there was. |
| 30. | Data and ﬁndings consistent | Was there consistency between the data presented and the ﬁndings? | Yes, there was. |
| 31. | Clarity of major themes | Were major themes clearly presented in the ﬁndings? | Yes, they were. |
| 32. | Clarity of minor themes | Is there a description of diverse cases or discussion of minor themes? | Yes, there is. |
